# Melatonin synergizes with prostaglandin E2 to enhance YAP-mediated regenerative epithelial cell emergence during intestinal repair

**DOI:** 10.1038/s41392-025-02248-1

**Published:** 2025-05-19

**Authors:** Yoojin Seo, Ji-Su Ahn, Hansong Lee, Yun Hak Kim, Hyung-Sik Kim

**Affiliations:** 1 Department of Oral Biochemistry, Dental and Life Science Institute, School of Dentistry, Pusan National University, Yangsan, South Korea; 2 Department of Anatomy, Pusan National University School of Medicine, Yangsan, South Korea

**Keywords:** Regeneration, Intestinal stem cells, Gastrointestinal diseases

**Dear Editor**,

Crypt base columnar cells (CBCs) are essential for the homeostatic renewal of the gut lining, yet they are susceptible to cytotoxic damage.^1^ In the absence of CBCs, alternative cell populations drive regeneration through fetal-like reprogramming and adaptive differentiation. In particular, a rare, slow-cycling cell lineage, referred to as revival stem cell (RSC), is activated by prostaglandin E2 (PGE_2_) secreted from the stromal niche, which subsequently stimulates the YAP pathway to facilitate the regeneration.^2,3^ Given the critical role of RSCs in intestinal recovery, regulating cellular plasticity to drive endogenous RSCs could serve as a powerful strategy for regenerative medicine.

In this context, we explore the role of melatonin, abundantly secreted by enteroendocrine cells (EECs) yet overlooked beyond its circadian regulation, in promoting intestinal regeneration. Notably, melatonin treatment suppressed proliferation in mouse small intestinal organoids without compromising viability (Fig. 1a), as evidenced by growth resumption after melatonin withdrawal (data not shown). Next, we utilized FUCCI reporter organoids to monitor cell-cycle dynamics. Melatonin-exposed IOs exhibited a rapid transition from the S/G2/M phase (green) to the G0/G1 phase (red) (Fig. 1a). Tracking of G0 cells with a mutant p27-mVenus fusion protein also revealed a marked increase in p27-expressing cells in the melatonin-treated group (Fig. 1a), suggesting that melatonin induces quiescence in IOs.

**Fig. 1:**
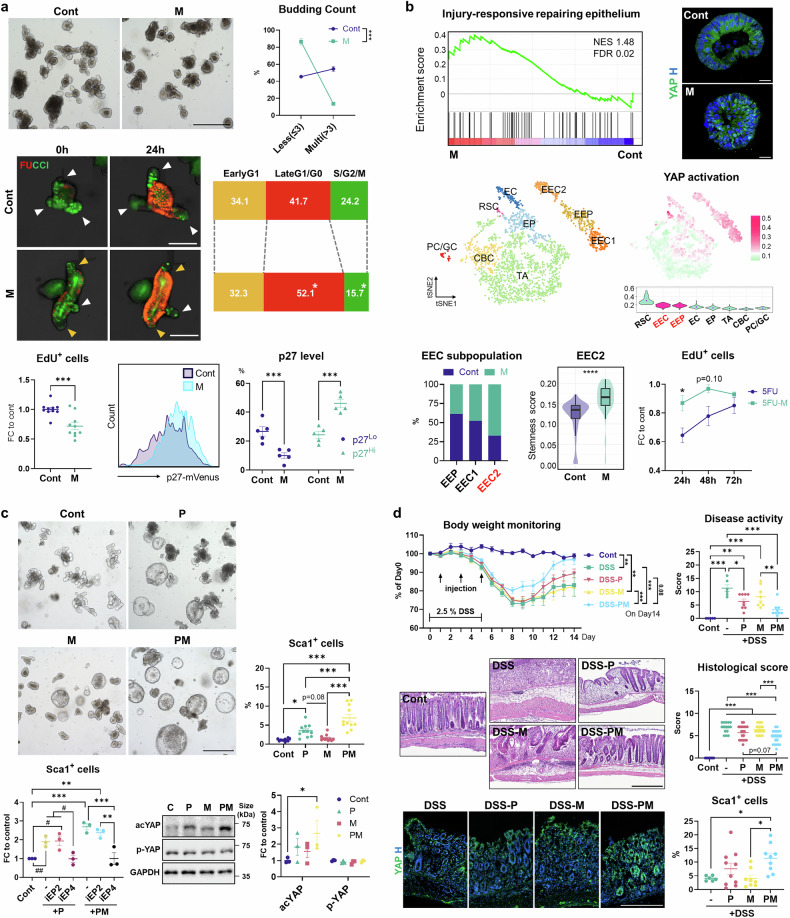
The role of melatonin in facilitating intestinal regeneration. **a** Melatonin triggers a dormant-like status in IOs. (Top) Bright-field images of control and melatonin-treated organoids, along with quantitative analysis of their budding counts (less: ≤3 buds; multi: >3 buds). (Middle) Cell cycle dynamics in control and melatonin-treated IOs were analyzed using the FUCCI reporter system. In the time-lapse fluorescence images, white arrowheads indicate proliferating regions, while yellow arrowheads mark quiescent parts. (Bottom, left) The proportion of EdU^+^ proliferating cells in the melatonin-treated group is presented as a fold change relative to controls. (Bottom, middle and right) The quiescent populations in organoids are visualized using a p27-mVenus reporter, with corresponding flow cytometry histograms and dot plots depicting p27 expression changes. **b** Melatonin treatment induces regenerative features accompanied by YAP activation in IOs. (Top, left) GSEA plot showing significant enrichment of injury-responsive epithelial gene set in melatonin-treated IOs. (Top, right) Immunofluorescence staining of YAP in control and melatonin-treated IOs. (Middle, left) t-SNE plot from scRNA-seq data of control and melatonin-treated IOs, illustrating cell distribution based on cellular identities. (Middle, right) Enrichment scores for regeneration-associated pathways are mapped onto the t-SNE plot, with violin plots showing their scores across different cell clusters. (Bottom, left) Bar graph showing the relative proportions of EEC subpopulations in control and melatonin-treated groups, alongside violin plots illustrating stemness scores within the EEC2 population. (Bottom, right) Assessment of proliferative capacity in 5-FU–exposed IOs with or without melatonin post-treatment, based on EdU incorporation. **c** Melatonin treatment accelerates PGE_2_-mediated RSC induction in IOs. (Top) Bright-field images of IOs treated with PGE_2_ and/or melatonin alongside dot plots depicting their Sca1⁺ RSC proportions, as determined by flow cytometry analysis. (Bottom, left) Flow cytometry-based quantification of Sca1^+^ cells in IOs treated with either PGE_2_, EP2 agonist (butaprost), EP4 agonist (Cay10598), an EP2 inhibitor (PF-04418948), or an EP4 inhibitor (L-161,982), with or without melatonin. (Bottom, right) Representative Western blot image and corresponding quantification of YAP levels in IOs treated with PGE_2_ and/or melatonin. **d** Administration of melatonin and PGE_2_ confers protection against DSS-induced colitis. (Top) A graph of daily body weight monitoring and a dot plot of disease activity scores on day 14. (Middle) Representative H&E-stained colon sections of DSS-treated mice with corresponding histological scoring results. (Bottom, left) Immunohistochemistry (IHC) of YAP in the colon on day 7. (Bottom, right) A dot plot showing the proportion of cryptic Sca-1-expressing cells on day 14, as determined by flow cytometry. The number of biological replicates corresponds to the number of dots on the graph. Scale bars = 500 μm (**a**, Top), 50 μm (**a**, Middle), 20 μm (**b**), 500 μm (**c**), 200 μm (**d**, Middle) and 300 μm (**d**, Bottom). Data are shown as the mean ± SEM and compared by unpaired *t*-test (**a**, **b**) or One-way ANOVA with Tukey’s multiple comparisons test (**c**, **d**), while Two-way ANOVA with Tukey’s multiple comparisons was used for body weight monitoring results (**d**). *P < 0.05, **P < 0.01, ***P < 0.001, ****P < 0.0001. In (**c**) and (**d**), #*P* < 0.05, ##*P* < 0.01 and ###*P* < 0.001, where the statistical significance was determined by unpaired *t*-test

To illustrate the transcriptomic changes in IOs upon melatonin treatment, we performed microarray analysis. According to gene set enrichment analysis (GSEA), gene set associated with injury-responsive repairing epithelium was significantly enriched in IOs upon melatonin treatment (Fig. 1b). Regenerative responses are accompanied by a fetal-like reversion of the non-ISC population in a manner dependent on YAP signaling.^4,5^ Intriguingly, gene expression patterns in melatonin-treated organoids closely mirrored those of fetal-derived IOs, with a positive correlation to the YAP activation-featured genes (data not shown). Indeed, melatonin treatment increased total YAP expression in IOs and promoted its nuclear translocation (Fig. 1b). These data underscore the potent role of melatonin in reinforcing YAP activation in IOs.

Next, we performed single-cell RNA sequencing (scRNA-seq) analysis to characterize cell-type-specific signatures in IOs altered by melatonin. Principal component analysis followed by t-SNE was used to visualize organoid-derived single-cell transcriptomes, revealing distinct clusters corresponding to known intestinal epithelial lineages based on marker gene expression (Fig. 1b). Melatonin decreased the CBC proportion while increasing enterocytes and their progenitors (EC/EP) and EECs in IOs, with no significant changes in the Ly6a^+^ RSC population (data not shown). To identify the subpopulation responsible for the enhanced regenerative features in melatonin-treated IOs, we visualized the enrichment patterns of transcriptomes relevant to intestinal repair. Notably, alongside the RSC population, EEC clusters exhibited transcriptomic signatures of YAP activation, injury-responsive epithelium and fetal spheroid (Fig. 1b and data not shown). The EECs comprise two sub-clusters, with melatonin treatment particularly promoting the emergence of the EEC2 (Fig. 1b). CytoTRACE analysis evaluating cellular differentiation states revealed that EEC2 cluster demonstrated significantly higher scores upon melatonin exposure, which contributed to an overall upregulation in the stemness of the EEC population (Fig. 1b). These results suggest that melatonin promotes a shift toward enhanced stemness in the intestinal epithelium, partly by fostering a subset of EECs poised to support regeneration. To test this, we examined whether melatonin aids organoid repair. Following 5-fluorouracil (5-FU) treatment, organoids collapsed, showing a marked reduction in EdU-incorporating cells. In contrast, post-treatment of melatonin facilitated the reparative response to 5-FU toxicity in IOs, leading to increased proliferation and budding restoration (Fig. 1b). These data indicate that melatonin reprograms intestinal epithelial cells, promoting their regenerative potentials and facilitating repair.

To investigate how melatonin intervenes in the intestinal reparative process, we evaluated its effects relative to PGE_2_, a well-known inducer of RSCs.^2^ Compared to PGE_2_ treatment alone, the combined treatment of PGE_2_ and melatonin (PM) significantly promoted the transformation of organoids into spheroids, suggesting that melatonin potentiates the effects of PGE_2_ (Fig. 1c). While melatonin alone minimally influenced RSC induction, as demonstrated by scRNA-seq results, PM treatment significantly increased the emergence of Sca-1^+^ cells, far exceeding the impact of PGE_2_ (Fig. 1c). This synergistic effect of PM on RSC induction was replicated in organoids treated with melatonin and the PGE_2_ receptor 4 agonist (aEP4) but not with the EP2 agonist (aEP2) (Fig. 1c). Similarly, disruption of EP4 signaling (iEP4) mitigated spheroid formation and reduced the frequency of Sca1^+^ cells in both the PM− and PGE_2_− treated group, whereas the EP2 inhibitor had no appreciable impact (Fig. 1c). PGE_2_-EP4 signaling activates the YAP pathway to drive the intestinal regenerative process.^2^ Interestingly, PM treatment robustly increased the abundance of activated YAP protein in IOs compared to the PGE_2_-treated group (Fig. 1c). Thus, melatonin enhances PGE_2_ signaling through the EP4 receptor, thereby reinforcing YAP activation.

Finally, we explored the therapeutic potential of PGE_2_ and melatonin in mitigating intestinal injury using a dextran sulfate sodium (DSS)-induced colitis model. Notably, PM group showed remarkable improvements in phenotypic monitoring parameters, with substantial epithelial restoration surpassing that of other groups, as confirmed by histological analysis (Fig. 1d). Considering the PM treatment led to the mildest weight loss and fastest recovery following colitis induction, we conducted a time-course analysis of intestinal YAP signaling to illustrate the regenerative responses. Upon DSS exposure, YAP activity gradually increased, with particularly high levels observed in the PGE_2_-treated mice. Interestingly, the PM group exhibited an earlier onset of nuclear translocation of YAP in the injured crypt region than others (Fig. 1d). Moreover, a trend of increased Sca-1⁺ cells in DSS-exposed crypts following PGE_2_ administration was further potentiated by melatonin co-treatment (Fig. 1d), reflecting the synergistic role of melatonin and PGE_2_ in inducing the RSC population.

In conclusion, our findings demonstrate that melatonin can be integrated into the PGE_2_-EP4-YAP signaling axis to facilitate injury-responsive reprogramming and RSC induction in the intestinal epithelium both in vitro and in vivo. Our study reveals a previously unappreciated role of melatonin as a pro-regenerative modulator, highlighting its potential to enhance intestinal repair and paving the way for novel therapeutic strategies in regenerative medicine.

## Supplementary information

Sigtrans Supplementary Materials

Dataset 3

Dataset 2

Dataset 1

## Data Availability

The data and materials used in this study are available from the corresponding authors upon reasonable request. The scRNA-seq data reported in this study is available from Gene Expression Omnibus with accession code GSE293340.
