# Supplementary material for: Melatonin synergizes with prostaglandin E2 to enhance YAP-mediated regenerative epithelial cell emergence during intestinal repair
Source: Signal Transduct Target Ther. 2025 May 19;10:163. doi: 10.1038/s41392-025-02248-1 (PMC12086221; doi:10.1038/s41392-025-02248-1)
Supplement: Supplementary file 1 — Sigtrans Supplementary Materials [file 41392_2025_2248_MOESM1_ESM.docx]

Supplementary Materials for

Melatonin synergizes with prostaglandin E2 to enhance YAP-mediated regenerative epithelial cell emergence during intestinal repair

Yoojin Seo†, Ji-Su Ahn†, Hansong Lee†, Yun Hak Kim^*^, and Hyung-Sik Kim^*^

Correspondence to: [hskimcell@pusan.ac.kr](mailto:hskimcell@pusan.ac.kr) ; [yunhak10510@pusan.ac.kr](mailto:yunhak10510@pusan.ac.kr)

**This PDF file includes:**

Materials and methods

Reference for supplementary materials

**Other Supplementary Materials for this manuscript include the following:**

Data set 1-3

**Materials and methods**

***Intestinal organoid culture, chemical treatment and image-based analysis***

Murine IOs from the small intestine were cultured as previously described.^1^ For tracking cell cycle status or p27 expression patterns, organoids were transfected with lentivirus containing FUCCI reporter (a gift from Kevin Brindle & Duncan Jodrell; Addgene plasmid #86849) or a p27-mVenus reporter (a gift from Mohamed Bentires-Alj; Addgene plasmid #176651),^2^ following established protocols.^3^ Chemical treatment was conducted as follows: melatonin (500μM), dm-ProstaglandinE_2_ (500nM), Butaprost (20μM), Cay10598 (10μM), L-161,982 (10μM), and PF-04418948 (10μM). Organoid images were captured with BioTek Cytation 5 Cell Imaging Multi-Mode Reader (Agilent, Santa Clara, CA) and processed with Gen5 software.

***Library preparation, preprocessing and analysis of scRNA-seq data***

Single cells from three independent organoid lines were pooled for the control- and melatonin-treated group on culture day 3. Single-cell RNA-seq libraries were prepared using the 10x Genomics Chromium Single Cell 3’ v3 platform and sequenced on the Illumina NovaSeq 6000. Raw data were processed with Cell Ranger v3.1.0 and analyzed using Seurat v4. Cells with 1,000–6,000 detected genes and <15% mitochondrial gene content were retained. Features expressed in ≥0.1% of cells were included. Data were log-normalized, and variable features were selected using the vst method. Dimensionality reduction was performed using PCA followed by t-SNE, and clustering was conducted using the DBSCAN algorithm. Cluster-specific marker genes were identified with the ‘FindAllMarkers’ function, with DEGs defined by Bonferroni-adjusted p < 0.05. We then characterized the clusters by comparing these DEGs with canonical intestinal lineage markers.^6-8^ Stemness was estimated using transcriptional diversity scores calculated with CytoTRACE v0.3.3.^9^ Pathway enrichment scores were obtained by averaging expression of gene sets and visualized on t-SNE plots using Seurat’s ‘FeaturePlot’ function. The gene lists for regeneration-related pathways were as listed in Data set 1. The DEG of each cluster is provided in Data set 2.

***Animal models for dextran sodium sulfate (DSS)-induced colitis and evaluation***

To induce experimental colitis, 2.5% DSS (w/v) was administered to 9-week-old C57BL/6 mice from day 0 via drinking water for 5 days, while the control group (Cont) received regular drinking water. The DSS-treated mice were divided into four experimental groups (n=10 per group): PBS, Melatonin (M), PGE_2_ (P), and PGE_2_ + Melatonin (PM). PGE_2_ (0.1 μg/g) and/or melatonin (7 μg/g) were dissolved in 200 µL of PBS and administered via intraperitoneal injection on days 1, 3, and 5. Disease activity index (DAI) scoring on day 14 and histological evaluation with H&E-stained colon sections were conducted as previously described.^1^ The daily statistics of body weight monitoring results, individual scoring results for DAI and histological assessment are provided in Data Set 3.

***Protein extraction and western blot***

Western blotting was performed as previously described. ^1^ Used primary antibodies were: active anti-YAP (1:1000), anti-phospho-YAP (1:1000) and anti-GAPDH (1:2000) (All from Cell Signaling Technology).

***Flow cytometry (FC) analysis***

FC analysis of IOs was performed as previously described. ^1^ To evaluate the proliferative capacity, organoids were incubated with a 10μM EdU solution for 2 hours and dissociated into single cells, followed by assessment with Click-iT EdU FC Assay Kit (Thermo Fisher Scientific). To perform FC analysis with murine intestine samples, ~5cm of ileum tissues were dissociated with buffer (10mM EDTA, 3% FBS in PBS) at 37°C. Obtained single cells were blocked with FcR blocking reagent (Miltenyi Biotec, Germany) and stained with antibodies for 1 hour at 4°C. To selectively capture the RSC population within intestinal epithelial cells, CD45^-^ TER-119^-^ CD31^-^ and EpCAM^+^ cells were gated, and their Ly6a/Sca-1 expression was evaluated. Samples were analyzed using the BD Accuri™ C6 Plus (BD Biosciences).

***Immunohistochemistry (IHC) and immunofluorescence (IF)***

IHC and IF were performed as previously described^1^ with anti-YAP (1:250) (Cell Signaling Technology). Subsequent imaging was acquired with Leica TCS confocal microscope (Leica Microsystems) or BioTek Cytation 5 Cell Imaging Multi-Mode Reader (Agilent).

***Statistical analysis***

Results are presented as mean ± SEM. The number of biological replicates was described in the figure legend. All statistical analyses as indicated in each figure legend were performed using GraphPad Prism 9 software (GraphPad Software, Inc., La Jolla, CA). For the scRNA-seq data, we used the Wilcoxon rank sum test in R to assess the statistical significance between groups.

**References**

1 Regmi, S. *et al.* Heterospheroid formation improves therapeutic efficacy of mesenchymal stem cells in murine colitis through immunomodulation and epithelial regeneration. *Biomaterials* **271**, 1-14 120752 (2021).

2 Ishikawa, K. *et al.* Identification of Quiescent LGR5(+) Stem Cells in the Human Colon. *Gastroenterology* **163**, 1391-1406 e1324 (2022).

3 Andersson-Rolf, A., Fink, J., Mustata, R. C. & Koo, B. K. A video protocol of retroviral infection in primary intestinal organoid culture. *J Vis Exp*, e51765 (2014).

4 Hao, Y. *et al.* Integrated analysis of multimodal single-cell data. *Cell* **184**, 3573-3587 e3529 (2021).

5 Hahsler, M., Piekenbrock, M. & Doran, D. dbscan: Fast Density-Based Clustering with R. *J Stat Softw* **91**, 1-30 (2019).

6 Haber, A. L. *et al.* A single-cell survey of the small intestinal epithelium. *Nature* **551**, 333-339 (2017).

7 Ayyaz, A. *et al.* Single-cell transcriptomes of the regenerating intestine reveal a revival stem cell. *Nature* **569**, 121-125 (2019).

8 Fawkner-Corbett, D. *et al.* Spatiotemporal analysis of human intestinal development at single-cell resolution. *Cell* **184**, 810-826 (2021).

9 Gulati, G. S. *et al.* Single-cell transcriptional diversity is a hallmark of developmental potential. *Science* **367**, 405-411 (2020).
